# Intermediate and long-term AKI outcomes in a public health system

**DOI:** 10.1007/s40620-025-02367-6

**Published:** 2025-08-01

**Authors:** Ana Muñoz-Sánchez, Leyre Martín-Rodríguez, Paula López-Sánchez, Maria Valdenebro, Maria Luisa Serrano-Salazar, Maria Marques, Jose Portoles

**Affiliations:** 1 Hospital Universitario Puerta de Hierro-Majadahonda, IDIPHISA, C/Manuel de Falla S/N, Majadahonda, 28222 Madrid, Spain; 2 Instituto de Investigación Puerta de Hierro-Segovia Arana, IDIPHISA, Madrid, Spain; 3 Medicine department Facultad de Medicina Universidad Autónoma de Madrid, Madrid, Spain

**Keywords:** Acute kidney injury, Chronic kidney disease, Dialysis, Mortality, Kidney replacement therapy

## Abstract

**Background:**

Acute Kidney Injury (AKI) is frequent and is associated with adverse outcomes.

**Aims:**

To analyze the impact of community-acquired and hospital-acquired AKI on in-hospital and five-year post-discharge kidney replacement therapy (KRT) requirement in the pre-COVID era.

**Methods:**

We linked the regional health system database of 419,851 admissions to the regional KRT registry. We grouped all admissions into 3 categories: community-acquired AKI, where AKI was the primary diagnosis, and hospital-acquired AKI, where AKI was an additional diagnosis alongside another primary condition. Admissions without this code were grouped into a third category (no AKI). We excluded patients aged under 18 years old, those with previous KRT, and pregnant women. The study was approved by the ethics committee. Patients were followed up for five years after discharge.

**Results:**

Community-acquired AKI accounted for 0.6% of all admissions, associated prolonged average hospital stays, and increased mortality rates. In-hospital KRT administration was required in 3.1% of cases, and after a mean follow-up time of 459 days, 7.2% of these patients began chronic KRT. Hospital-acquired AKI represented 6.1% of all admissions and was associated with the highest mortality rate (22.9% vs 14.4% in the community-acquired AKI group) and the longest average hospital stay (12.6 days vs 7.1 in the no AKI group). Only 0.5% of hospital-acquired AKI cases required KRT during the AKI episode, while 2% of these patients initiated chronic KRT after a mean follow-up time of 594 days.

**Conclusions:**

AKI continues to be a frequent problem in clinical practice, negatively influencing patient morbidity and mortality, and increasing the risk of starting KRT in the medium-long term.

**Graphical abstract:**

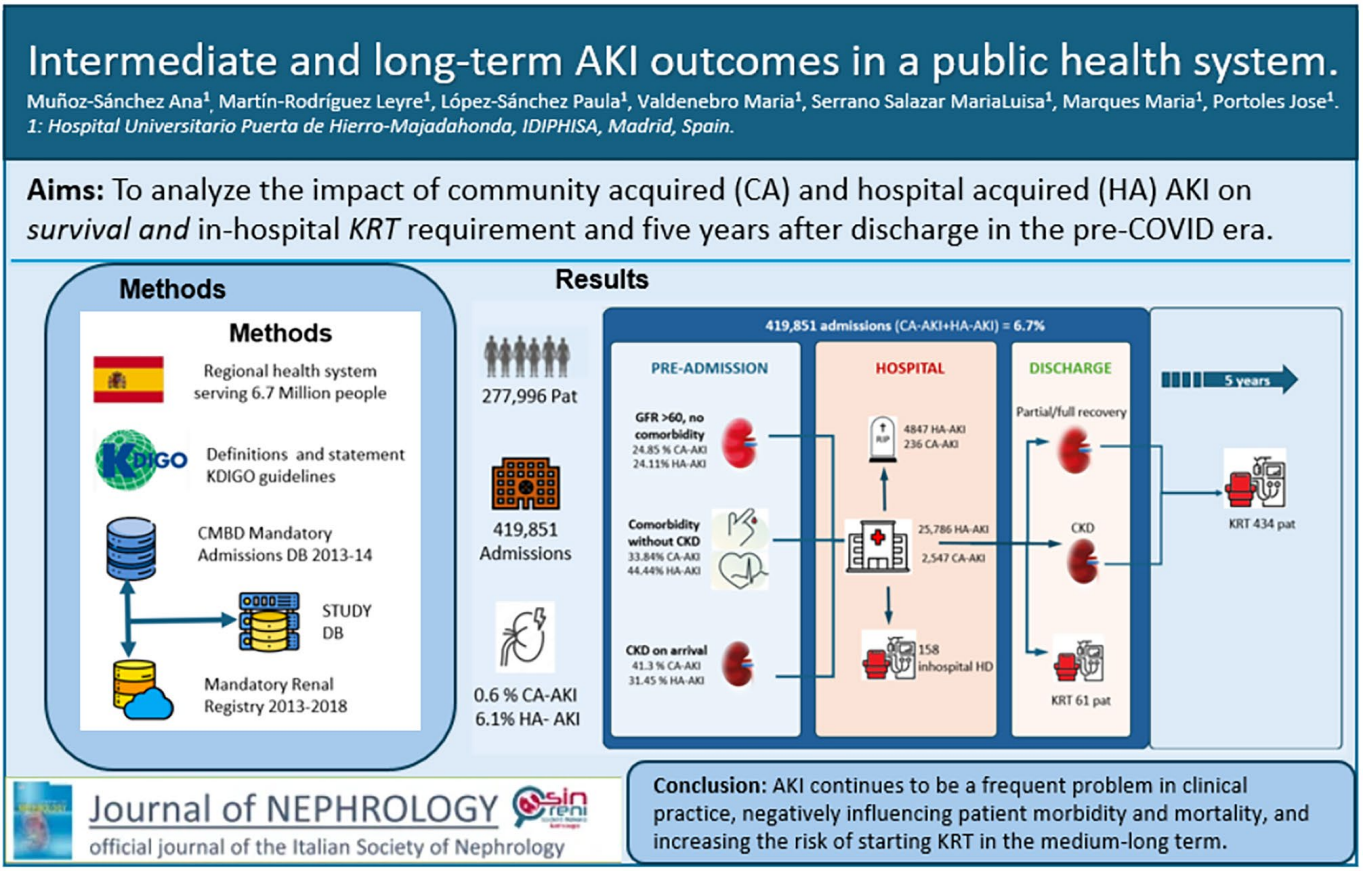

## Introduction

Acute Kidney injury (AKI) continues to be a relevant clinical issue that leads to increased morbidity and mortality, predisposes patients to chronic kidney disease (CKD) [1] and is associated with a notable rise in healthcare costs [2]. In the United States, hospital-acquired AKI is associated with cost increases ranging from $5.4 to $24 billion [2, 3].

Variations in climate, ethnicity, culture, socioeconomic status and health system can affect AKI etiology and management [4]. Hospital-acquired AKI incidence, according to several meta-analyses, is around 22.3% in North America, while it fluctuates between 19.3% and 25.2% in Europe [4]. Limitations in coding accuracy hinder the correct interpretation of AKI.

AKI risk factors are well-identified, but their potential consequences are not tracked over time. There are few studies that follow patients long-term after kidney failure, assess the need for kidney replacement therapy (KRT) during AKI, or examine subsequent entry into kidney replacement therapy.

One of the “Healthy People 2020” goals was to increase the percentage of AKI cases followed by nephrology [5], since even mild kidney failure with full recovery of kidney function is associated with a greater than 40% risk of developing stage 3 CKD or higher, compared to patients without AKI [6]. Mortality rates increase with AKI severity, from 15.9% in stage 1, to 47.8% in stage 3, to 49.3% in AKI requiring dialysis.

The Madrid AKI study group GEFRAM [7] evaluated all AKI episodes in referral hospitals in our regional health system over a 9-month period. Quiroga et al. [8], analyzed AKI mortality with 30-day follow-up, including 1720 admissions, however, both studies lack further long-term follow-up.

The concept of Acute Kidney Disease (AKD) has emerged to describe the phase following an AKI episode, which can last from 7 to 90 days [9–11]. This term allows us to consider kidney disease as a continuum: AKI-AKD-CKD-KRT [9]. The Adequate Dialysis Quality Initiative (ADQI) Workgroup emphasizes the need to improve transitional care after AKI to mitigate long-term effects. However, data on effective interventions to improve AKI or AKD outcomes remain limited [9].

Available studies are heterogeneous or involve short follow-up times [12]. Additionally, temporal and regional comparisons of AKI incidence and prognosis may be biased by changes in knowledge, monitoring, recognition, documentation, and AKI clinical practice [5].

Our study aims to provide an objective perspective that includes the public healthcare system of Madrid (SERMAS), with universal coverage; establish protective strategies during AKI, and expand our horizons in its management.

## Methods

This is a retrospective study that includes all patients admitted between 2013 and 2014 to eight reference hospitals in the regional health system serving 6.7 million people, linked with a follow up from the Madrid Registry of Renal Patients (REMER) over the subsequent 5 years. Data were analyzed prior to the pandemic to avoid the potential bias of COVID-19 on acute kidney failure [13, 14].

Data were obtained from the Minimum Basic Data Set (MBDS) of the public Health Service. The MBDS was integrated with the REMER database, which collects all patients in chronic KRT in the Autonomous Community of Madrid. This is a mandatory database which must be completed for all centers caring for KRT patients [15].

From the source data, secondary variables were generated using validated algorithms, such as the Charlson Comorbidity Index and in-hospital mortality risk estimators, based on previous publications [16]. All information was pseudonymized.

Admissions related to childbirth and/or cesarean sections (ICD-9 code 540/541/542/560) were excluded. Minors under 18 years of age and patients with prior KRT before the diagnosis of AKI were also excluded.

All patients were followed up for 5 years after discharge, with data linked using MBDS identification codes and the REMER database.

Demographic data and information related to the admission were collected, including whether the reason for admission was a surgical procedure and the associated mortality risk. Readmissions within 30 and 180 days due to the same AKI, as well as those within 180 days due to a new AKI, were also recorded.

### Definitions

The unit of analysis was the admission, regardless of whether it was associated with an AKI episode. Admissions were grouped by patient only for readmission analysis or Cox regression models. Admissions were categorized based on the presence of an acute kidney failure diagnosis in the MBDS (ICD-9 code 584); two subgroups were established based on whether AKI was the primary reason for admission: community-acquired AKI where AKI was the primary diagnosis, and hospital-acquired AKI where AKI was an additional diagnosis alongside another primary condition. Admissions without this code were grouped into a third category (no AKI).

Time to event was defined as the period from the first AKI admission to the initiation of dialysis, death, or the end of follow up (December 31, 2018). Readmissions were defined as admissions occurring within the same diagnostic category within 30- and 180-days post-discharge to estimate recurrence (percentage and time).

Readmissions due to a new AKI episode were also identified.

### Statistical analysis

We present data using descriptive statistics, including the mean and standard deviation (SD), median and interquartile range (IQR), or percentages. Group comparisons were conducted using Student's *t*-test, ANOVA, or Chi-square tests depending on the type and distribution of the variables. The outcomes analyzed included mortality and dialysis requirement during admission. We also evaluated the number of patients who remained on KRT at discharge and those who started chronic dialysis during a 5-year follow-up period. Additionally, we assessed the risk factors for AKI and its prevalence within our regional health system.

Kaplan–Meier survival curves were used to estimate time to dialysis initiation, and were compared using the log-rank test. Risk factors for developing hospital-acquired AKI were estimated using logistic regression, excluding cases of community-acquired AKI from the analysis. Risk factors for initiating KRT before discharge or for experiencing in-hospital mortality were also identified using a logistic regression model. To determine risk factors for initiating dialysis after hospital discharge, a Cox regression analysis was performed. For multivariate analysis, we included variables with a *p* < 0.1 in the univariate model and those deemed clinically relevant.

The analyses were conducted using Stata Statistical Software version 15 (College Station, TX: StataCorp LLC) [17].

This study is part of a project authorized by the REMER Registry Committee and by the Ethics Committee for Clinical Research (CEIm) of Hospital Puerta de Hierro Majadahonda (ref 132/16) [16]. A waiver of informed consent was obtained for this study.

## Results

**Fig. 1:**
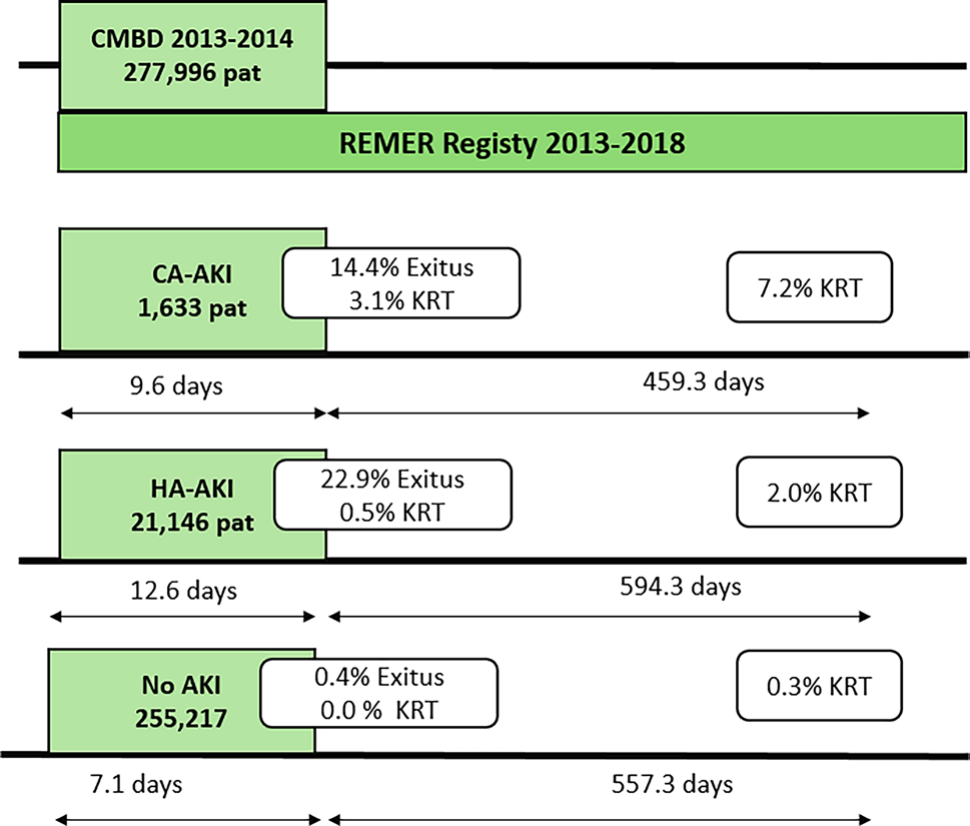
Patient flow chart and clinical outcomes (Exitus and KRT). *KRT* Kidney replacement therapy, *AKI* Acute kidney injury, *CA* Community acquired, *HA-AKI* Hospital-acquired. *Pat*: Patients

**Fig. 2:**
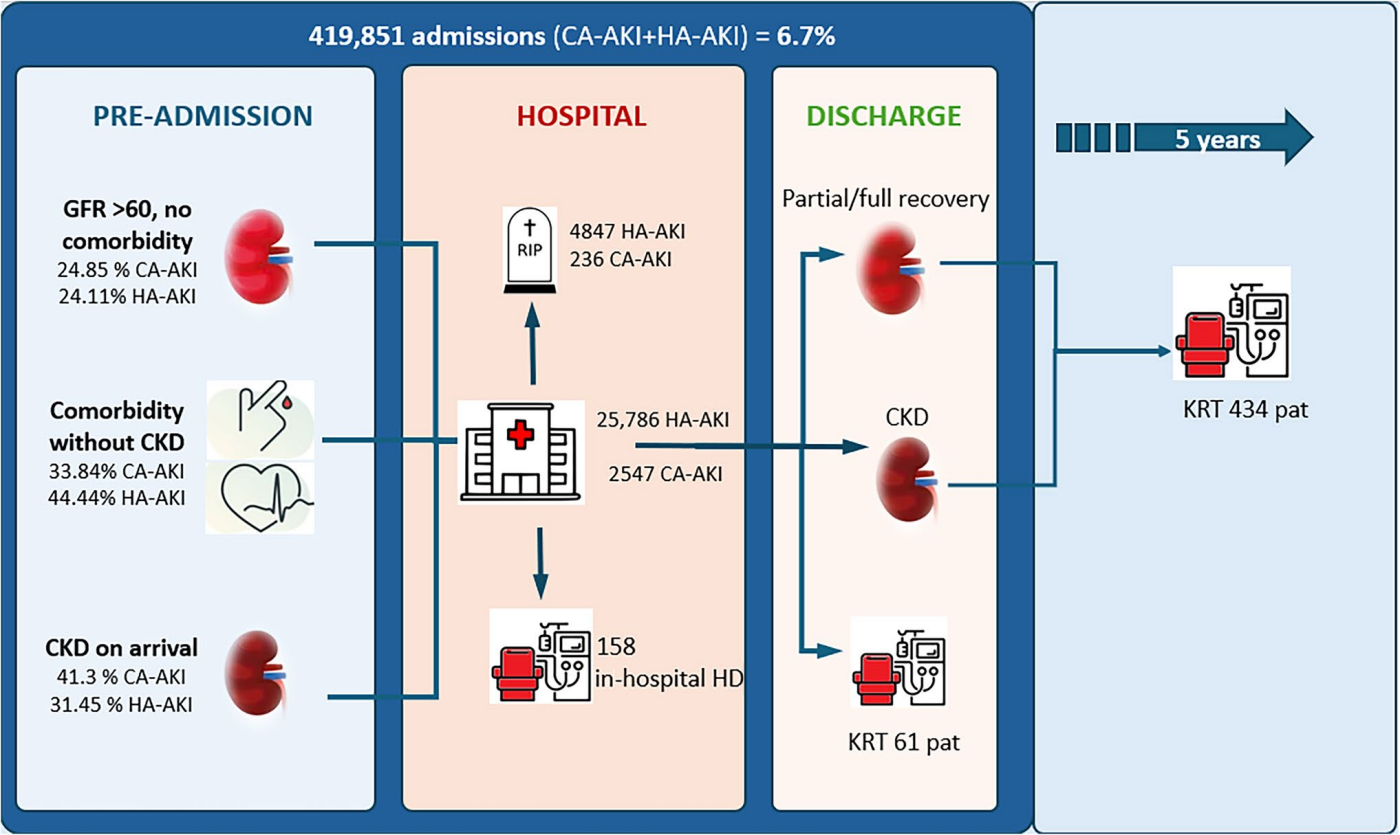
The continuum of acute and chronic kidney disease. *CKD* Chronic kidney disease, *KRT* Kidney replacement therapy, *AKI* Acute kidney injury, *CA* Community acquired, *HA-AKI* Hospital acquired, *GFR* Glomerular filtration rate, *pt*: patients

Our study includes 277,996 patients admitted between 2013 and 2014, who were followed for five years until 2018 (Fig. 1). Of the total admissions (419,851), 34.6% of patients had pre-existing comorbidities without CKD, and 6.7% had CKD. After discharge, 61 patients remained on dialysis, and during the 5-year follow-up period, 434 patients initiated KRT (Fig. 2);

Patients were grouped into three categories: community-acquired AKI, hospital-acquired AKI, and no AKI. The characteristics of each group are summarized in Table 1.

**Table 1:** Characteristics of admitted patients grouped into CA-AKI, HA-AKI, vs no-AKI

	No AKI	CA-AKI	HA-AKI	All	*P* value
N (%)	391,518	2547	25,786	419,851	
	93	0.6	6.1	100	
Age (y; mean, SD)	63.6 (19.0)	74.8 (15.2)	77.9 (13.7)	64.6 (19.0)	< 0.001
Male (%)	49.5	55.7	55.6	49.9	< 0.001
Charlson Comorbidity Index (median, IQR)	1 [0–2]	3 [1–4]	3 [1.4]	1 [0–2]	< 0.001
Diabetes mellitus (%)	17.1	34.1	30.4	18.0	< 0.001
CKD (%)	4.9	41.3	31.5	6.7	< 0.001
Unplanned admission (%)	57.4	94	92.5%	59.8	< 0.001
Surgical procedure (%)	44.7	4.4	16.6	42.7	< 0.001
Department (%)	IM 17.4 GS 11.9 TRA 9.6	IM 37.4 NEPH 34.1 ONC 4.4	IM 42.7 CAR 9.0 DIG 7.0		
Mortality risk (%)					< 0.001
High	12.3	28.9	55.5	15	
Very high	2.4	4.5	24.8	3.8	
OUTCOME					
In-hospital death (%)	3.5	9.3	18.8	4.5	< 0.001
Dialysis during admission (%)	0.1	8.4	3.9	0.4	< 0.001
Readmission at 30 d (same clinical pathology, %)	5.9	4.2	5.2	5.8	< 0.001
Readmission at 180 d (same clinical pathology, %)	14.6	11.5	14.9	14.6	< 0.001
Readmission due to AKI at 180 d (%)	-	8.9	6.9	0.5	< 0.001
KRT onset (%)	0.50	9.0	2.6	0.7	< 0.001

*CKD* Chronic kidney disease, *SD* Standard deviation, *IQR* Interquartile range, *d* days, *KRT* Kidney replacement therapy, *AKI* Acute kidney injury, *CA* Community acquired, *HA* Hospital acquired, *y* years, *IM* Internal medicine, *GS* General surgery, *NEPH* Nephrology, *ONC* Oncology, *TRA* Traumatology, *DIG* Digestive, *CAR* Cardiology

Focusing on the 28,333 patients with AKI, 41.3% of community-acquired AKI and 31.45% of hospital-acquired AKI had pre-existing CKD, while 33.84% of community-acquired AKI and 44.44% of hospital-acquired AKI admissions were patients with diabetes mellitus or a cardiovascular event without pre-existing CKD.

Community-acquired -AKI accounted for 0.6% of all admissions in our regional health system. This group experienced prolonged average hospital stays and increased mortality rates. In-hospital KRT was required in 3.1% of community-acquired AKI cases, and after a mean follow-up time of 459 days, 7.2% of these patients began chronic KRT, most of whom during the first year after discharge (59% of the community-acquired AKI group), showing a progressively decreasing trend from the second year onwards (Fig. 1).

**Fig. 3:**
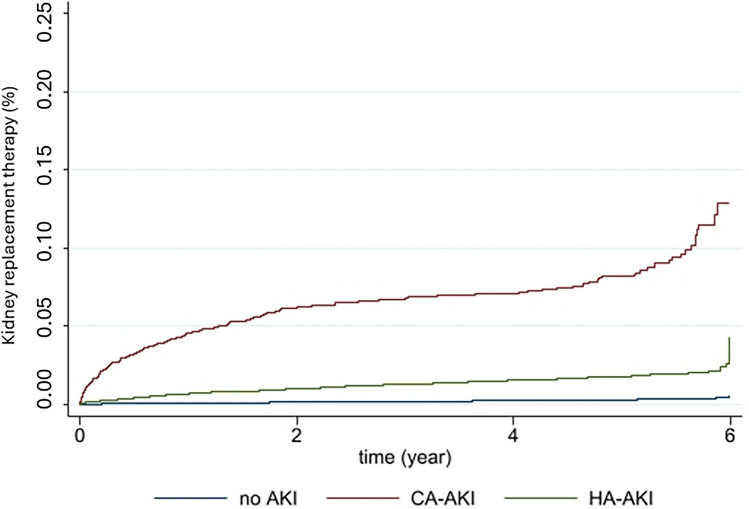
Post-discharge time of the “start of KRT”. Kaplan–Meier curve for kidney replacement therapy during follow-up. *KRT* Kidney replacement therapy, *AKI* Acute kidney injury, *CA* Community acquired, *HA-AKI* Hospital-acquired

The hospital-acquired AKI group represented 6.1% of all admissions and was associated with the highest mortality rate (22.9% vs. 14.4% in the community-acquired AKI group) and the longest average hospital stay (12.6 days vs. 7.1 days in the no AKI group). Only 0.5% of hospital-acquired AKI cases required KRT during the AKI episode, while 2% of these patients initiated chronic KRT after a mean follow-up time of 594 days, most of whom during the first year after discharge (38.5% of hospital-acquired AKI) (Fig. 1). The Kaplan–Meier analysis of the time to chronic KRT is shown in Fig. 3. Patients discharged after community-acquired AKI showed a trend toward a higher cumulative incidence of events, although this did not reach statistical significance (*p* value log-rank < 0.001).

The main risk factor for hospital-acquired AKI is prior CKD (OR: 3.6, CI 95% [3.48–3.74]), which remains significant after adjusting for age, sex, and comorbidity (Table 2). A sub-analysis (multivariate analysis 2) focusing on patients without prior CKD, demonstrates that risk factors such as age, comorbidity, and being male are also associated with an increased risk of developing hospital-acquired AKI.

**Table 2:** Risk models for evaluating Hospital-Acquired AKI (Multivariable models)

	Multivariate1 (R^2^ 16.4%)		Multivariate2 (R^2^ 10.4%)	
	OR	CI 95%	OR	CI 95%
Previous CKD	3.61	[3.48–3.74]	-	
Age 1st quartile (ref)	1.0		1.0	
2nd quartile	1.95	[1.83–2.08]	1.95	[1.82–2.1]
3rd quartile	2.97	[2.79–3.15]	3.03	[2.83–3.24]
4th quartile	5.0	[4.71–5.31]	5.58	[5.23–5.95]
Female	0.83	[0.81–0.85]	0.76	[0.74–0.79]
Charlson Comorbidity Index 1st quartile (ref)	1.0		1.0	
2nd quartile	1.82	[1.73–1.92]	1.77	[1.68–1.86]
3rd quartile	2.2	[2.09–2.31]	1.98	[1.88–2.08]
4th quartile	2.91	[2.78–3.05]	2.38	[2.27–2.5]
Surgical procedure	0.41	[0.4–0.43]	0.40	[0.38–0.42]

*CKD* Chronic kidney disease, *OR* Odds ratio, *CI* Confidence interval

Regarding in-hospital mortality, 22.9% of patients with hospital-acquired AKI died, compared to 14.4% in the community-acquired AKI group and 5.4% in those without AKI. Mortality risk in community-acquired AKI is 1.5 times, and in hospital-acquired AKI 3.5 times, that of an admission without AKI, even after adjusting for age, sex, comorbidity, and type of admission. Lower mortality risk was observed for surgical admissions (Table 3).

**Table 3:** Multivariable risk models to evaluate kidney replacement therapy (KRT) onset and mortality

	Multivariate1		Multivariate2	
	OR	CI 95%	OR	CI 95%
Outcome: KRT onset	R^2^ 8.6%		R^2^ 25.2%	
AKI group				
No-AKI (reference)	1.0		1.0	
CA-AKI	35.7	[25.5–50.1]	7.5	5.3–10.7
HA-AKI	6	[4.5–7.8]	1.7	1.3–2.3
Charlson Comorbidity Index (with age)	1.1	[1.1–1.2]	0.8	0.8–0.9
Female	0.4	[0.3–0.6]	0.5	0.4–0.6
Surgical procedure	1.6	[1.2–2.0]	2.1	1.7–2.7
Previous CKD	–	-	119.2	82.6–172.1
Outcome: Death	R^2^ 15.4%		R^2^ 16.0%	
AKI group				
No-AKI (reference)	1.0		1.0	
CA-AKI	1.5	[1.4–1.8]	1.9	[1.7–2.2]
HA-AKI	3.5	[3.3–3.6]	4.1	[4.0–4.3]
Charlson Comorbidity Index (with age)	1.3	[1.3–1.3]	1.3	[1.3–1.3]
Female	1.1	[1.0–1.1]	1.0	[1.02–1.08]
Surgical procedure	0.4	[0.4–0.5]	0.4	[0.41–0.44]
Previous CKD	–	–	0.5	[0.4–0.5]

*CKD* Chronic kidney disease, *KRT* Kidney replacement therapy, *AKI* Acute kidney injury, *CA* Community acquired, *HA* Hospital acquired, *OR* Odds ratio, *CI* Confidence interval

The logistic regression model indicates that the risk of initiating KRT after an AKI event is substantially elevated: 35.7 times higher CI 95% [25.5–50.1] for community-acquired AKI patients and 6.0 times higher CI 95% [4.5–7.8] for hospital-acquired AKI patients compared to those without AKI. These findings hold even after adjusting for age, sex, comorbidity, and type of admission, with a lower risk observed for surgical admissions (Table 3).

Patient follow-up enabled an analysis of risk factors for KRT onset within a five-year period. Starting KRT was more likely in patients with prior CKD and in those without prior CKD who had relevant risk factors such as diabetes or previous cardiovascular events. In multivariate analysis 2, (excluding CKD), community-acquired AKI emerged as the strongest predictor of KRT initiation (Table 4).

**Table 4:** Risk models to evaluate kidney replacement therapy (KRT) onset at 5 years

	Univariate	Multivariate1	Multivariate2
	HR [CI 95%]	HR [CI 95%]	HR [CI 95%]
Previous comorbidity			
No CKD or Comorbidity (reference)	1.0	1.0	1.0
previous CKD	77.2 [64.9–91.8]	164.5 [136.6–198.0]	NA
previous CV + DM	3.6 [2.9–4.4]	5.9 [4.8–7.3]	4.2 [3.3–5.2]
Age (y)	1.004 [1.002–1.007]		
Age stratification			
< 65 y (reference)	1.0	1.0	1.0
65–75 y	1.7 [1.5–1.9]	0.6 [0.5–0.6]	0.8 [0.6–1.0]
> 75 y	1.0 [0.9–1.1]	0.14 [0.12–0.16]	0.3 [0.2–0.4]
AKI group			
No AKI (reference)	1.0	1.0	1.0
CA-AKI	25.3 [21.1–30.5]	3.9 [1.2–4.7]	14.9 [8.5–26.0]
HA-AKI	5.8 [5.1–6.5]	1.4 [1.2–1.6]	6.3 [4.8–8.2]

*HR* Hazard ratio, *NA* Not applicable, *CKD* Chronic kidney disease, *CV* Cardiovascular, *DM* Diabetes, *CA* Community acquired, *HA-AKI* Hospital-acquired, *AKI* Acute kidney injury, *y* years, *CI* Confidence interval

## Discussion

Our study updates the incidence and risk factors of AKI in a hospital setting, highlighting its impact on mortality and the need for KRT in both the short and long term. This approach aims to reduce uncertainty regarding the integrated process of AKI-AKD-CKD-KRT, enabling a better understanding of its impact and facilitating the design of targeted strategies to improve the patient journey.

Both hospital-acquired and community-acquired AKI are associated with longer hospital stays, a higher risk of chronic KRT, and increased mortality. Risk factors for developing hospital-acquired AKI include pre-existing kidney disease, which remains a significant risk factor even after adjusting for age, sex, and comorbidity.

We have described the time course toward chronic KRT in patients who survive AKI and are discharged without being placed on dialysis. Patients with community-acquired AKI have a numerically higher cumulative incidence of chronic KRT initiation over the 5 years of follow-up than those with hospital-acquired AKI, and even higher than the rest of discharged patients. This information is highly relevant and is not adequately reported in dialysis and transplant registries, which do not consider AKI as a cause for dialysis initiation or as a triggering factor [18, 19].

Our analysis was based on mandatory databases that encompass all patient admissions within our regional healthcare system, which serves nearly seven million people. This comprehensive coverage ensures the internal validity of our findings, which could be extrapolated to similar healthcare systems.

The reported incidence of AKI varies widely across studies, primarily due to differences in AKI definitions, variations in study design, and differing clinical perspectives (e.g., nephrologists versus other specialists).

In 2017, the ADQI published a consensus document introducing the concept of acute kidney disease [10], emphasizing the concept of an *AKI-AKD-CKD continuum* [9]. However, the nature and prevalence of AKD have not been widely explored [20]. It is well recognized that one of the most significant risk factors for AKI is pre-existing CKD. Moreover, AKI itself is a major risk factor for the development of CKD and the progression of pre-existing CKD, thus closing the circle that links acute and chronic kidney failure, as we attempt to describe here. It is also important to note that AKD occurs in one in four survivors of AKI [10, 21], making its proper identification crucial to preventing further kidney disease progression.

In our study, hospital-acquired AKI accounted for 6% of all hospital admissions, with approximately three out of ten patients having pre-existing CKD. Sawhney et al. [5] reported that one out of seven hospitalized patients experienced AKI. They estimated an incidence of 17.6% for hospital-acquired AKI in patients with pre-existing kidney disease, compared to 8.4% in patients with normal kidney function. In Hodgson’s study [22], hospital-acquired AKI represented 8.3% of all admissions, compared to 7.3% for community-acquired AKI.

The incidence of AKI is increasing by 10% annually, with associated morbidity and mortality remaining high [21]. A multicenter study in China [23] developed a predictive model for in-hospital mortality and dialysis requirements in AKI patients. This model proved useful for identifying high-risk patients, improving mortality outcomes, and preventing the development of complications.

AKI risk factors described in the literature [3] include age over 65, male sex, diabetes, CKD, anemia, and heart failure. Among the predisposing factors, sepsis, major surgery, and nephrotoxin exposure are commonly noted. Pereira et al. [24] assessed the role of comorbidities in long-term mortality following AKI. They observed that mortality after an AKI episode was more strongly associated with pre-existing comorbidities than with the severity of the episode itself.

Sykes et al. [12] refer to “AKI care bundles,” as educational packages designed to better address AKI and potentially prevent its occurrence. One of the goals of “Healthy People 2020” was to increase the percentage of AKI cases managed by nephrology [5].

However, only 8% of patients who experience AKI requiring dialysis are followed by a nephrologist after discharge [25]. Moreover, delays or the absence of nephrology assessment are associated with higher mortality, dialysis dependence, and prolonged length of stay [4]. Unfortunately, nephrology evaluation is not always available, which underscores the importance of establishing a multidisciplinary working group, designing protective strategies, and implementing a shared care plan during hospitalization and after hospital discharge [26].

Requiring dialysis during AKI may be necessary (around 3% in the community-acquired AKI group), which is consistent with the literature (ranging from less than 5% [3] to 11% [4]); however, only a minority remain dependent on KRT after discharge. KRT initiation is more likely in patients with pre-existing CKD or in those without CKD but with significant cardio-metabolic comorbidities (diabetes mellitus or cardiovascular diseases). A prior AKI episode is also a risk factor. Rimes et al. [27] observed that fewer than 10% of survivors of an AKI episode required dialysis after 5 years, which aligns with our results (around 7% in community-acquired AKI).

The “0 by 25” initiative of the International Society of Nephrology aims to prevent all avoidable deaths from AKI worldwide by 2025 [4, 5].

AKI mortality was higher in the hospital-acquired AKI group. A systematic review published in 2009 [28] reported that mild AKI was associated with a 70% increased risk of mortality, and the risk of long-term mortality was nearly three times higher in patients with moderate-to-severe AKI compared to patients without AKI.

Another systematic review [29] identified AKI as an independent risk factor for CKD, kidney failure, and mortality. Patients with AKI had higher risks of developing CKD (HR 8.8, 95% CI 3.1–25.5), kidney failure (HR 3.1, 95% CI 1.9–5.0), and mortality (adjusted HR 2.0, 95% CI 1.3–3.1) compared to patients without AKI.

The association between AKI and mortality has been widely described. Sawhney et al. [5] observed that the more severe the AKI, the higher the short-term mortality, with the risk persisting at discharge, particularly in the first-year post-hospitalization. Ten-year mortality was higher among those with AKI and severe baseline CKD (eGFR < 30 mL/min/1.73 m^2)^). This study also identified previous AKI episodes as an additional adverse prognostic factor in those with AKI.

Heung et al. [6] reported an AKI-associated mortality rate between 25–50%, which is somewhat higher than what we found in our study. They concluded that patients with hospital-acquired AKI had a substantial risk of developing CKD in the year following hospitalization, and the timing of AKI recovery was a strong predictor, even for mild AKI forms. One of the most surprising findings of this study was that mild AKI with rapid recovery (< 2 days) was associated with > 40% risk of developing stage 3 or greater CKD compared to patients without AKI.

Establishing recovery patterns would be crucial to stratify patients at risk. In this regard, the ADQI guidelines [3] published a consensus to establish five recovery categories after AKI. Ronco et al. [26] found a higher survival rate in the early recovery group (> 90% at 1 year), compared to 40% survival in the non-recovery group. Relapses were associated with a five-fold increase in the risk of death at 1 year.

Patients with recurrent kidney failure or AKD have higher mortality rates and greater rates of readmissions [20]. Up to 20% of AKI patients are readmitted within 30 days of discharge [30]. In our study, readmission rates were around 4–5%. Neyra et al. [21] reported recurrence rates of AKI in 25% of patients who survived an AKI event, with readmission occurring in up to 50% of patients who experienced AKI in the year following discharge.

Holmes et al. [30] examined the impact of recurrent AKI episodes. They included all AKI events in elderly patients between April 2015 and September 2018. Of these, 29.3% experienced a second episode, 9.3% a third, and 4% experienced four or more episodes. For patients who survived, failure to recover kidney function after any AKI episode was significantly associated with a higher likelihood of experiencing further kidney failure.

CKD secondary to AKI carries a higher 6-month mortality risk than CKD due to other causes [21]. Further investigations are needed to expand our understanding, particularly regarding the etiology of AKI (sepsis, ischemia–reperfusion, drugs) [11], and whether this can modify its course, prevent relapses, or promote recovery [26].

In light of all the above, we propose an approach to kidney disease as a continuum, advocating for protocolized AKD follow-up at 3 months, and 1 year post-discharge at the very least, to anticipate CKD progression [31] and potential KRT requirement. We also suggest documenting AKI as a cause of kidney failure and conducting further studies to expand our knowledge in this subgroup.

The present study should be considered in terms of its limitations and strengths. This observational retrospective study was conducted in a regional health system, but it involved almost 7 million people.

We do not have data on estimated glomerular filtration rate (eGFR) at discharge or during the follow-up. Therefore, our study is unable to distinguish complete or partial renal recovery, nor to stratify AKI stages; however, by focusing on the continuum with kidney replacement therapy, it specifically targets AKI stage 3. We cannot analyze the mortality of those discharged without dialysis requirements, but we do provide data on in-hospital mortality and the mortality of those who initiated chronic KRT. For both reasons, there remain areas of uncertainty within the overall AKI-AKD-CKD-KRT continuum model. Nevertheless, the study benefits from the strength of including all AKI cases and all patients starting chronic KRT, regardless of the admitting service or direct nephrology team involvement.

## Conclusions

AKI is associated with longer hospital stays, higher mortality rates, and an increased risk of requiring kidney replacement therapy in the medium- to long-term.

For the first time, we have demonstrated the influence of AKI on the incidence of KRT over the medium to long term, and have reduced the level of uncertainty in the general AKD-CKD-KRT model. We propose several areas for improvement in this care pathway, including post-discharge nephrological follow-up, the inclusion of AKD history in patient records, and a comprehensive analysis of the AKD-CKD-KRT process. Only through these efforts can we develop specific protocols and interventions to reduce the long-term impact of AKI.

## Data Availability

Data will be provided upon request to the corresponding author.
